# Development of Grouting Materials from Shield Sludge via Alkaline Hydrothermal Activation: A Resource Utilization Approach

**DOI:** 10.3390/ma18122673

**Published:** 2025-06-06

**Authors:** Lianjun Chen, Meiyue Liu, Penghui Li, Junxiang Wang, Xiaoqiang Cao

**Affiliations:** 1 College of Safety and Environmental Engineering, Shandong University of Science and Technology, Qingdao 266590, China; skdclj@sdust.edu.cn (L.C.); lipenghui@sdust.edu.cn (P.L.); 2 School of Mechanical & Automotive Engineering, Qingdao University of Technology, Qingdao 266520, China; 3 College of Energy and Mining Engineering, Shandong University of Science and Technology, Qingdao 266590, China; 4 Institute of Yellow River Delta Earth Surface Processes and Ecological Integrity, Shandong University of Science and Technology, Qingdao 266590, China

**Keywords:** shield sludge, synchronous grouting, alkaline hydrothermal, hydration product

## Abstract

Frequently, the viscous mixture from shield operations is disposed of because its significant water ratio and the presence of polymers like foaming agents result in subpar structural qualities, contributing to the unnecessary consumption of land and the squandering of soil assets. Therefore, these problems urgently need to be solved economically and effectively. This study relies on the shield sludge produced by Qingdao Metro Line 6 project, and sand and shield sludge were used as the raw materials for synchronous grouting. By applying the basic principles of geopolymerization, ingredients like shield sludge and ground granulated blast furnace slag (GGBS) were mixed with sodium hydroxide, serving as the activating agent, in the preparation of the simultaneous grout formulas. A broad range of laboratory tests was conducted to evaluate the performance of these grout formulations. The effects of varying material ratios on key performance indicators—namely, fluidity, water secretion rate, setting time, and 3-day unconfined compressive strength (UCS)—were systematically analyzed. Based on these findings, the optimal material ratios for shield sludge-based synchronous grouting materials were proposed. Subsequently, component geopolymer was prepared from the activated shield sludge and shield sludge without adding any additional alkaline activators by simply adding water. A geopolymer with a 28-day compressive strength of 51.08 MPa was obtained when the shield sludge dosing was 60 wt%. This study aims to provide a reference for the preparation of synchronous grouting materials for the resource utilization of shield sludge.

## 1. Introduction

Shield construction has the advantages of high construction accuracy, little damage to the urban environment, fast construction speed, and more, and has become the most commonly used method in tunnel construction [1,2,3]. However, with the continuous construction and planning of subway and underground tunnel projects, a large amount of shield sludge is being generated [4,5]. At present, China generates over 50 million cubic meters of shield tunneling slurry annually, but its utilization rate is less than 5% [6]. Because water and various additives are usually added during shield construction to reduce friction resistance and improve excavation speed, the engineering performance of shield sludge is poor. Therefore, these sludges cannot be directly used in engineering practice and are usually discarded, resulting in a waste of resources [7,8,9].

In view of the above problems, many scholars have conducted extensive research on the reuse of shield sludge, mainly involving the fields of building materials, filling materials, and grouting materials [5,10,11,12]. In terms of building materials, researchers mainly focus on extracting sand and gravel with specific properties from shield sludge as raw materials for concrete and mortar [13]. Because the shield sludge often contains clay minerals and organics, especially in the clay layer, the common practice is to convert a large number of shield sludge into building products such as bricks, ceramics and blocks through the firing process [14,15,16]. In terms of filling materials, according to the particle size distribution of shield sludge, an appropriate method for the reuse of residues can be determined. The coarse-grained residue can be effectively utilized in specialized applications such as subgrade, cushion, and foundation filling, but only after undergoing pretreatment processes like multistage crushing and grading adjustment [17,18,19]. In terms of grouting materials, the sand and clay in the shield sludge are selectively used to replace the sand aggregate and bentonite in the synchronous grouting slurry, and the main components include cement, quicklime, fly ash, bentonite, and sand in equal quantities. Among them, the resource utilization of shield sludge replacing part of synchronous grouting raw materials is one of the research hotspots in recent years [20,21].

The primary sources of synchronous grouting materials consist of commercially available precast grout and on-site produced grout made from purchased raw materials, such as sand aggregates and cementitious components. These materials represent a substantial portion, approximately 10%, of the total cost involved in shield tunneling slurry construction. Among synchronous grouting materials, the main source of the cementitious materials is ordinary Portland cement (OPC) [22]. Given the increasing environmental concerns and the need to reduce carbon emissions, there has been growing interest in developing sustainable alternatives to OPC [23,24]. However, its production process is characterized by high energy consumption (approximately 3 GJ per ton) and substantial CO_2_ emissions (around 800 kg per ton) [25]. These adverse impacts on resources and the environment have prompted extensive research to develop alternative eco-friendly cementitious materials and auxiliary binders. Geopolymer is an inorganic aluminosilicate material with an amorphous three-dimensional (3D) structure, exhibiting many excellent properties such as low cost, high environmental friendliness, high strength, high temperature resistance, and outstanding durability [26,27]. The most widely used aluminosilicate precursors in geopolymer synthesis include fly ash, slag, metakaolin, or their blends, due to their high reactivity under alkaline activation [28,29,30,31]. While sodium silicate/waterglass activators enhance gel formation in conventional two-part geopolymers, their hygroscopicity, high viscosity, and elevated SiO_2_/Na_2_O modulus trigger process instability, cost inefficiency, and premature gelation, critically limiting scalable engineering use.

This research utilized shield sludge as a primary raw material for the preparation of synchronous grouting material. Based on the geopolymerization principle, shield sludge and ground granulated blast furnace slag (GGBS) were employed as precursors, while sodium hydroxide (NaOH) was used as the chemical activator to synthesize the grouting material [32,33]. A comprehensive series of laboratory tests were conducted to assess the performance of the synchronous grouting material, focusing on key parameters such as consistency, fluidity, 2-h bleeding rate, setting time, and 3-day unconfined compressive strength (UCS). These tests allowed for the identification of the optimal material ratio for shield sludge-based synchronous grouting. This study provides a practical basis for the resource-efficient utilization of shield sludge.

## 2. Experimental Program

### 2.1. Testing Materials

The synchronous grouting material is composed of cementitious material and fine aggregate, supplemented by other modified materials and mixed in an appropriate proportion. A new type of synchronous grouting material was prepared using the shield sludge of Qingdao Metro Line 6. The formula uses shield sludge and GGBS as raw materials, sand and admixture, and sodium hydroxide as activator.

**Figure 1:**
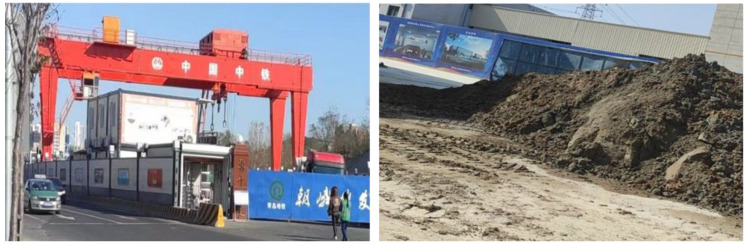
In situ sampling of shield sludge.

**Figure 2:**
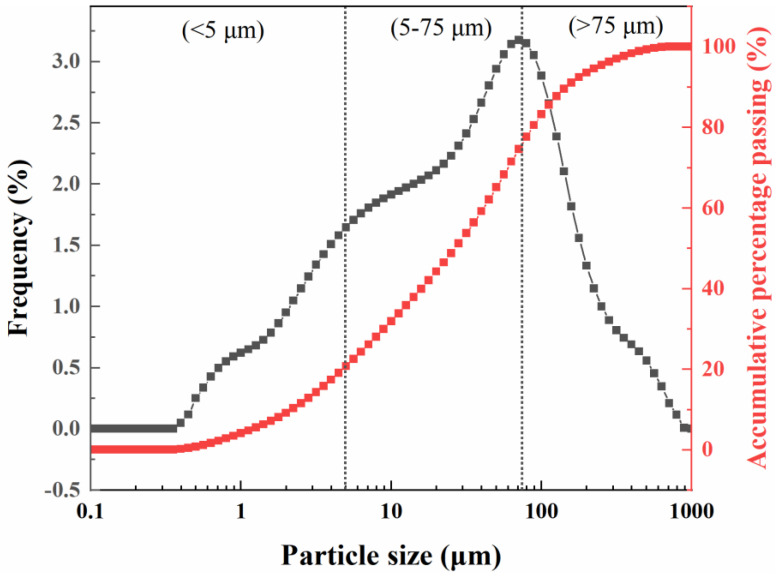
Particle size distribution curve of shield sludge soil.

Shield sludge soil: The slag soil discharged from the site is shown in Figure 1. The representative soil of Qingdao Metro Line 6 was analyzed by a laser diffraction granulometer (Microtrac, Montgomeryville, PA, USA). As shown in Figure 2, the overall particle size of the fine sludge soil is fine, with an average diameter of about 28.40 μm, of which the content of <5 μm fine particles is 22.40%, the content of 5–75 μm particles is 54.42%, and the content of >75 μm particles is 23.18%. The raw material was sieved using a 150 μm sieve to separate it into two fractions: the coarse fraction (>150 μm), which was used as coarse aggregate for concrete, and the fine fraction (<150 μm), which consisted of fine-grained sludge. The fine sludge was further treated using alkaline thermal activation technology to enhance its hydration reactivity.

Table 1 summarizes the oxide compositions of both the shield sludge and the commercially available Portland Composite Cement (P·C), as determined by X-ray fluorescence (XRF, Rigaku Ultima IV, Tokyo, Japan).

### 2.2. Experimental Scheme Design

To enhance the hydration reactivity of shield sludge, an alkaline hydrothermal activation method was employed to treat fine sludge with particle sizes below 75 μm. A solid-to-liquid ratio of 1:1 was used, mixing NaOH solution with the fine sludge, then pouring the mixture into a stainless steel autoclave and placing it in an oven for the reaction. The activation temperatures were 100 °C, 150 °C, 200 °C, and 250 °C for 4 h, and each sample was labeled as Te-100, Te-150, Te-200, and Te-250. The activation times were 2 h, 4 h, 6 h, and 8 h at 200 °C, and each sample was labeled as Ti-2, Ti-4, Ti-6, and Ti-8. After activation, the samples were removed from the autoclave and dried in a vacuum oven at 100 °C. The dried samples were then ground using a vibratory mill to a particle size of <75 μm, obtaining the activated sludge.

The laboratory temperature is maintained at (20 ± 2) °C. Firstly, the grouting material was prepared with activated residue and slag as the main raw materials, and the performance of the grouting material was controlled by changing the content of slag. The slag content was 20%, 40%, 50%, 60%, 70%, and 80% respectively, and each sample was recorded as Cm-20, Cm-40, Cm-50, Cm-60, Cm-70, and Cm-80, respectively. At the same time, the reference samples were prepared with the original residue, slag, and NaOH as raw materials under the same material ratio, and the samples were recorded as Co-20, Co-40, Co-50, Co-60, Co-70, and Co-80, respectively. The water-to-solid ratio of all samples was 0.6. After 1 day, the grout sample was demoulded. Then the grout specimens at different ages of 3 days, 7 days, and 28 days were cured in water at constant temperature (20 ± 2) °C.

### 2.3. Materials Characterization

An X-ray diffractometer (XRD, Rigaku Ultima IV, Cu Kα radiation, Tokyo, Japan) was used to obtain an X-ray diffractogram in the 2θ range of 5–70°, with a step size of 0.02° and a counting time of 0.75 s per step. At the same time, thermogravimetric analysis (TGA) was conducted on a Mettler Toledo TGA instrument (Mettler Toledo, Greifensee, Switzerland) at a temperature increase of 10 °C/min in a nitrogen atmosphere from 30 °C to 900 °C. Morphological and elemental analysis was carried out using a Zeiss SIGMA 300 field-emission scanning electron microscope (FE-SEM, Carl Zeiss AG, Oberkochen, Germany) equipped with an energy-dispersive X-ray spectroscopy (EDS) detector, operated at an accelerating voltage of 10 kV. The elemental composition of the samples and the specific concentration of the active metal were quantified using an ARL Quant’X energy-dispersive X-ray fluorescence (EDXRF) spectrometer (Thermo Fisher Scientific, Waltham, MA, USA) under vacuum conditions (≤10 Pa).

The preparation of paste specimens followed the Chinese standard GB/T 17671-2021 [34] Solid precursors were mixed with deionized water or NaOH solution using a planetary mixer for 5 min to ensure homogeneity. The fresh paste was then cast into 40 mm × 40 mm × 160 mm steel molds and subjected to curing in a controlled environment (20 ± 1 °C, relative humidity 90%). According to GB 175-2023 [35], compressive strength and hydration characteristics were assessed at curing ages of 1, 7, and 28 days. It is worth noting that potential carbonation may occur during both mixing and curing stages, which could influence the hydration process.

### 2.4. Testing Methodology for Bleeding Rate and Stone Formation Rate

A 250 mL graduated cylinder was placed on a level surface, and 245 mL ± 5 mL of freshly prepared synchronous grouting slurry was poured into the cylinder. After standing for 1 min, the initial level of the slurry (a_0_) was recorded, and the cylinder was sealed. After standing for 3 h, the level of the bleeding water (a_1_) and the level of the slurry (a_2_) were recorded. The bleeding rate (BR_3h_) was calculated using the following equation:

BR_3h_ = (a_1_ − a_2_)/a_0_ × 100 (1)

where

BR_3h_—bleeding rate after 3 h (%), accurate to 0.1%;

a_0_—initial slurry level (mL);

a_1_—level of bleeding water after 3 h (mL);

a_2_—level of slurry after 3 h (mL).

The level of the hardened slurry (a_3_) was also recorded after 3 h. The stone formation rate (HR_3h_) was calculated as follows:

HR_3h_ = a_3_/a_0_ × 100 (2)

where

HR_3h_—stone formation rate after 3 h (%), accurate to 0.1%;

a_3_—height of the hardened slurry after 3 h (mL).

## 3. Results and Discussion

### 3.1. Alkaline Hydrothermal Activation of Shield Sludge

The influence of sodium hydroxide concentration, activation temperature, and activation time on the crystalline structure and phase transitions of shield sludge was analyzed through X-ray diffractometry. Additionally, the morphological variations between the shield sludge and the activated slag were examined using scanning electron microscopy coupled with energy-dispersive spectroscopy. The optimum sodium hydroxide concentration was 20%, as described in previous studies.

#### 3.1.1. Effect of Activation Temperature

**Figure 3:**
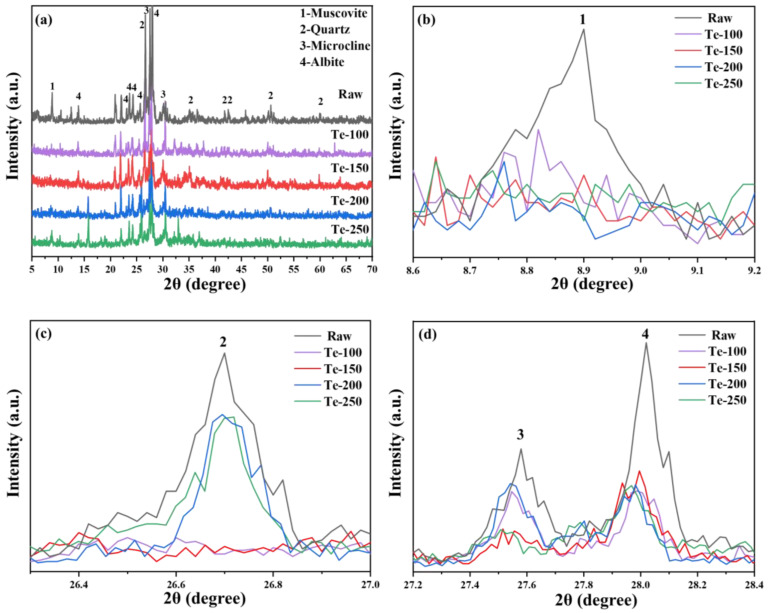
(**a**) XRD patterns of activated residue under different activation temperatures and enlarged images of the strongest diffraction peaks of (**b**) mica, (**c**) quartz, and (**d**) microcline and feldspar of activated slag at different activation temperatures.

The XRD patterns of activated residue at different activation temperatures are shown in Figure 3. Figure 3a shows that the diffraction peak intensities of major mineral phases such as mica (PDF# 73-1661), quartz (PDF# 79-1906), microcline (PDF# 26-0911), and feldspar (PDF# 80-1094), in the activated residue, are significantly lower than those in the original fine-grained residue [36,37]. Furthermore, as the activation temperature increases, the diffraction peak intensities of these mineral phases decrease even further. The results show that increasing the activation temperature is an effective strategy to reduce the relative crystallinity of residue in the process of alkali thermal activation. It is reported in relevant literature that after high-temperature alkali fusion treatment (>800 °C) of aluminosilicate raw materials, it is possible to observe the diffraction peaks of crystalline phases such as zeolite, sodalite, and nepheline in XRD patterns [38,39]. However, the diffraction peaks of these crystalline phases could not be found in the XRD spectra of activated slag, which is likely to be related to the lower alkali thermal activation temperature of slag. In addition, combined with the research results, it can be seen that amorphous sodium aluminosilicate is the main product of alkali thermal activation of residue, but it is difficult to identify it in XRD spectra due to its poor crystallinity [40].

**Figure 4:**
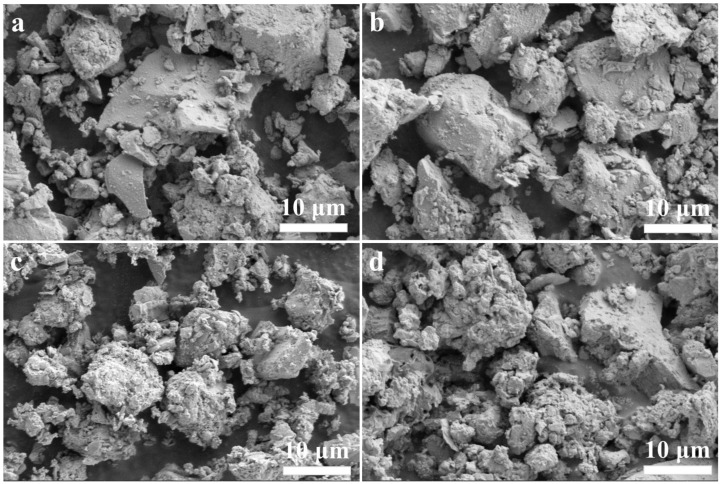
SEM images of shield sludge (**a**) Te-100, (**b**) Te-150, (**c**) Te-200, and (**d**) Te-250.

The SEM images of the activated muck samples Te-100, Te-150, Te-200, and Te-250 are shown in Figure 4. It can be seen that the activation temperature has a significant impact on the microscopic morphology of the muck. When the activation temperature is controlled at 100 °C, the edges and corners of large debris particles can be clearly observed in the sample Te-100, and the particle surfaces remain smooth, showing no signs of NaOH corrosion. At the same time, only a small amount of gel products can be found, piled into small particles, which are distributed in the gap between large residue particles [41,42]. The results show that at low temperature, the reaction degree between shield sludge and NaOH is low, and the shield sludge cannot be effectively activated, which is consistent with the high relative crystallinity of the Te-100 sample. With the activation temperature increasing to 200 °C and 250 °C, the reaction degree between shield sludge and NaOH is enhanced, and the shield sludge particles become coarser. A large number of gel products are formed and cover the surfaces of residue particles, making it difficult to observe the edges and corners of large residue particles. The change in micromorphology of residue particles at different activation temperatures again shows that increasing the temperature can effectively enhance the alkali thermal activation effect of residue.

**Figure 5:**
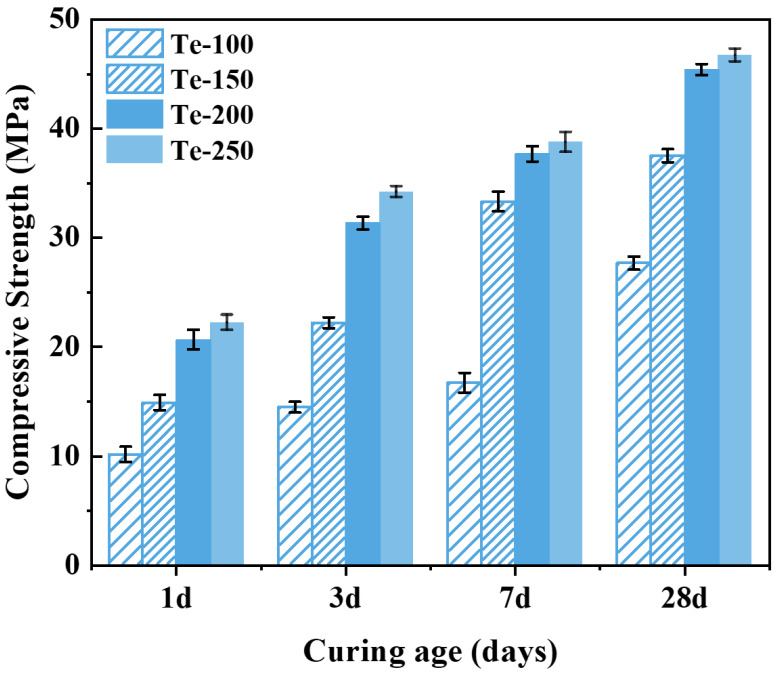
Effect of activation temperature on compressive strength of cementitious materials.

As can be seen from Figure 5, the compressive strength of the specimens showed a gradual increase with the increase in activation temperature. The compressive strength of specimen Te-100 was lower when the activation temperature was 100 °C, and its compressive strengths cured after 1, 3, 7, and 28 days were 10.18, 14.48, 16.73 and 27.68 MPa, respectively. The compressive strengths of the specimens increased significantly when the activation temperature was increased to 200 °C, and the compressive strengths of specimen Te-200 were 20.68, 31.35, 37.69, and 45.41 MPa, which are 103.14%, 115.81%, 125.28%, and 64.05% higher than those of specimen Te-100, respectively, and the enhancement is significant. This is because the increase in activation temperature improves the reaction degree between NaOH and the silica-aluminate phase in the shield sludge, and a large number of reactive ions such as Si^4+^ [6] and [Al]^3+^ can be released from the activated slag, which creates favorable conditions for the hydration reaction of the specimen [43,44]. However, as the activation temperature was further increased from 200 °C to 250 °C, the compressive strength of the specimens showed only a weak increase. Therefore, considering both performance enhancement and energy efficiency, an activation temperature of 200 °C is identified as the optimal condition for preparing cementitious materials from shield sludge.

#### 3.1.2. Effect of Activation Time

**Figure 6:**
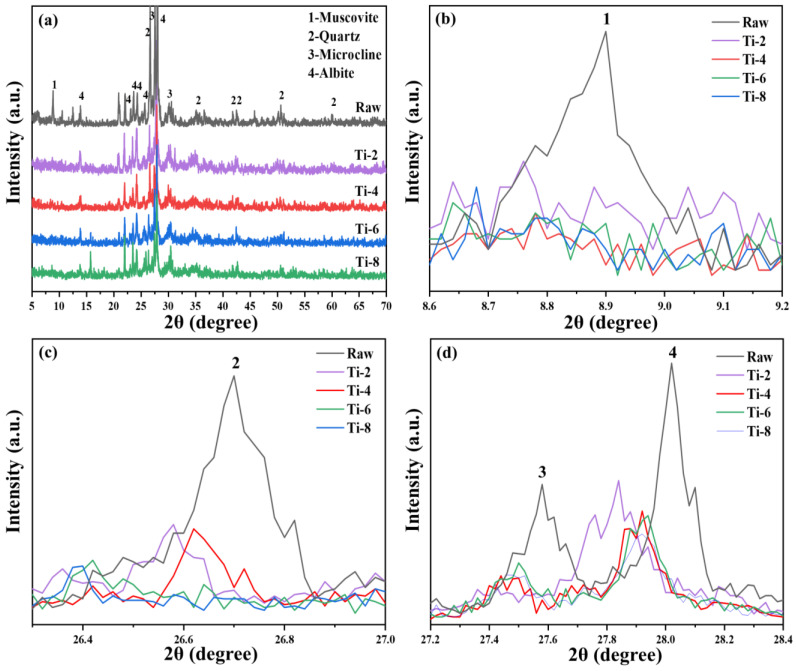
(**a**) XRD patterns of activated residue under different activation times and magnified images of the strongest diffraction peaks of (**b**) mica, (**c**) quartz, and (**d**) microcline and feldspar at different activation times.

The XRD patterns of activated residue under different activation times are shown in Figure 6. Compared with the original shield sludge, the diffraction peak intensities of mica, quartz, microcline, feldspar, and other major mineral phases in activated muck decreased significantly, and the longer the activation time, the weaker the diffraction peak intensities of the mineral phases. The results show that in the process of alkali thermal activation, prolonging the activation time can significantly reduce the crystallinity of the residue. Compared with the XRD of Ti-4, Ti-6, and Ti-8 samples, it can be seen that the diffraction peak intensities of mica, quartz, microcline, feldspar, and other mineral phases differ, and no new products can be found in any of the activated residue samples [45]. Combined with the previous research results, amorphous sodium aluminosilicate is the main product of alkali thermal activation of residue, but it is difficult to observe in XRD spectra due to its poor crystallinity [46].

**Figure 7:**
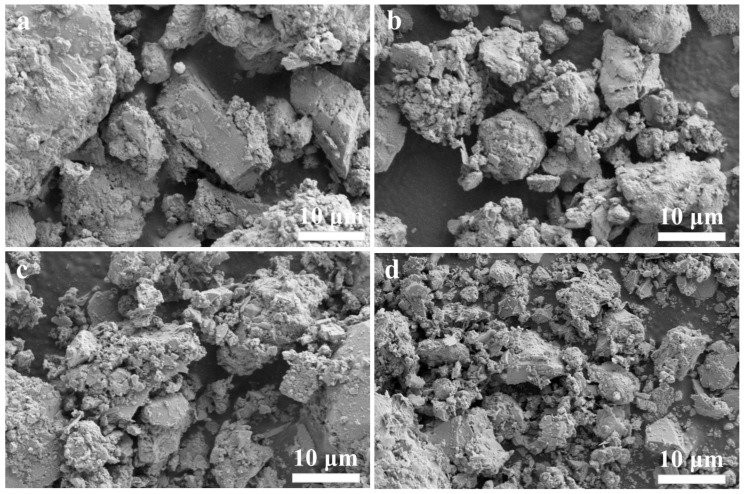
SEM images of the activated residue samples (**a**) Ti-2, (**b**) Ti-4, (**c**) Ti-6, and (**d**) Ti-8.

Figure 7 shows the SEM images of activation times 2, 4, 6, and 8 h. It can be seen that the activation time has a significant effect on the microstructure of shield sludge. When the activation times is controlled at 2 and 4 h, due to the reduced degree of reaction between the shield sludge and NaOH, the amount of gel-like products generated is less, which is not enough to completely encapsulate the shield sludge particles, and the angularity of some of the large shield sludge particles can still be observed in the specimen Ti-2 and Ti-4. The characteristics of the microscopic morphology of specimens Ti-2 and Ti-4 are consistent with their higher relative crystallinity. As the activation time was extended to 6 h and 8 h, the chemical reaction between the shield sludge and NaOH continued, and the activation degree of the shield sludge increased significantly compared with that of Ti-2. A large number of gel products can be observed to be generated and cover the surface of the shield sludge particles in specimens Ti-6 and Ti-8, and it is difficult to find the angularity of the large slag particles. However, due to the high activation degree of Ti-6 and Ti-8, the two microstructures do not show significant differences. The changes in the microscopic morphology of the clinker particles at different activation times again show that prolonging the activation time can effectively enhance the alkaline thermal activation effect of clinker [3,47].

**Figure 8:**
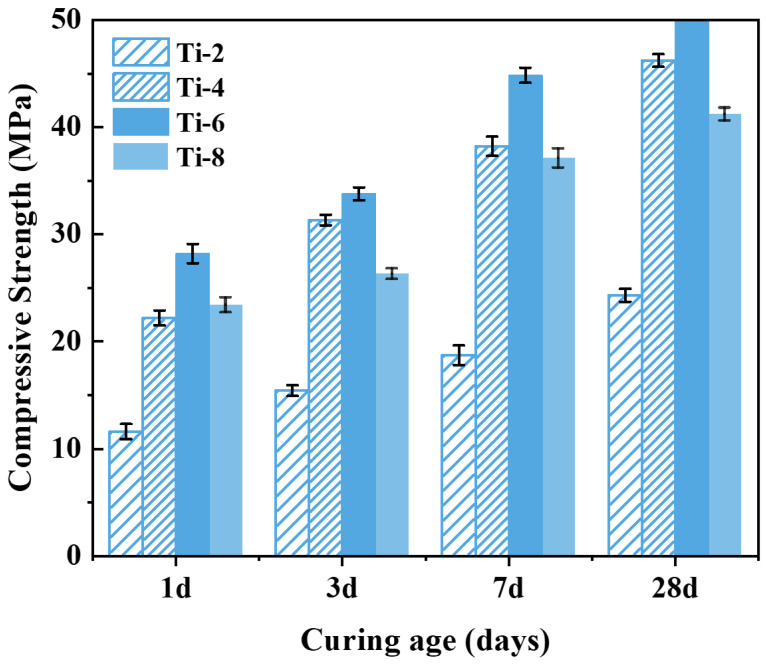
Effect of activation time on compressive strength of cementitious materials.

Figure 8 shows that the compressive strength tends to increase first and then decrease with the activation time. When the activation time is 2 h, the activation degree of the shield sludge soil is low at this time, and the leaching amount of active ions such as Si and Al is limited; thus, the compressive strength of specimen Ti-2 is low under this condition. With the activation time extended to 6 h, the chemical reaction between NaOH and the silica–aluminate phase in the residue continues, and a large number of Si, Al, and other active ions can be dissolved from the activated residue, greatly promoting the hydration reaction of the specimen. Under this condition, the compressive strength of specimen Ti-6 significantly increased, with compressive strengths at 1, 3, 7, and 28 days of curing of 28.20, 33.78, 44.84, and 50.39 MPa, respectively, which were 150.00%, 128.55%, 129.60% and 106.94% higher than those of specimen Ti-2, respectively. This indicates that an activation time of 6 h (Ti-6) provides the optimal balance between ion leaching and structural integrity, making it the best-performing combination among the tested conditions. However, as the activation time was further extended from 6 h to 8 h, the compressive strength of the specimens instead showed a clear decreasing trend. The alkaline activation reaction was nearly complete, as evidenced by the extensive coverage of shield sludge particles with gel-like products observed in SEM images (Figure 7c,d). Further prolongation of activation time did not enhance the dissolution of reactive Si and Al species, but likely promoted the formation of secondary crystalline or semi-crystalline phases (e.g., zeolite-like structures), reducing the availability of free ions and thereby inhibiting subsequent hydration reactions and strength development.

### 3.2. Analysis of Grouting Material Properties

When the shield tunneling machine digs in until the tube sheet is detached from the shield tail, the front and rear ends of the shield tunnel are unbalanced in force due to the injection of grouting material into the tunnel tube sheet, and at this time, the long setting time of the grouting behind the wall leads to low early strength, so it is vital to control the setting time of the slurry as well as the early strength.

**Figure 9:**
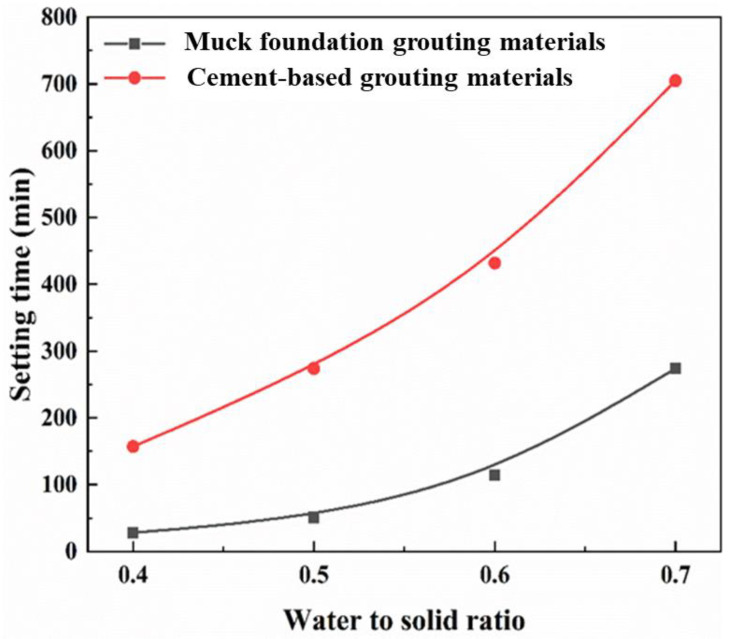
Setting time of different materials.

For comparison, the cement-based grouting material was prepared using P·C cement under identical conditions. The change in solidification time of grouting materials with different water–solid ratios is shown in Figure 9. When the water–solid ratio is 0.4, 0.5, 0.6, and 0.7, the setting time of shield sludge-based grouting material is 28, 51, 114, and 274 min, respectively, and that of cement-based grouting material is 157, 274, 432, and 705 min, respectively. The setting time of both shield sludge-based and cement-based grouting materials increases with the rise of the water–solid ratio. This is because higher water content dilutes the reactants, slows down the hydration reactions, and delays the formation of C–S–H gel. However, the shield sludge-based material still exhibits a much shorter setting time due to the presence of sodium hydroxide, which enhances the early hydration reaction and accelerates C–S–H formation.

### 3.3. Analysis of Water Secretion Rate and Stone Rate of Grouting Material

Slurry fluidity is one of the very important properties of grouting materials. It determines the viscosity and permeability of the slurry, and also determines the slurry grouting pressure, slurry volume, and many other factors.

According to Table 2, the control of the water–solid ratio is the main factor of the water secretion rate, and the influence of other factors is relatively small. From the figure, it can be seen that the larger the water–solid ratio is, the more water is added, and the greater the dilution effect on the slurry, resulting in increased spacing between shield sludge particles, and the fluidity becomes larger. As the water–solid ratio increases from 0.4 to 0.7, both the caking rate and the bleeding rate of the shield sludge-based grouting material and the cement-based material change accordingly. For the cement-based grouting material, the bleeding rate increases by 8.75%, exceeding the acceptable reference value when the water–cement ratio reaches 0.6. In contrast, the bleeding rate of the shield sludge-based grouting material remains at 0%. In general, the bleeding rate of both types of slurry shows a positive correlation with the water–solid ratio. However, the bleeding rate of the cement-based material is consistently higher than that of the shield sludge-based material. From a construction perspective, to maintain an acceptable bleeding rate, the water–solid ratio should be properly controlled. Specifically, for the shield sludge-based grouting material, the water–solid ratio should not exceed 0.6. The stone formation rate of grouting materials decreases with increasing water–solid ratio. For cementitious grouting materials, the rate drops below the reference value of 95% when the water–solid ratio reaches 0.7. In contrast, shield sludge-based grouting materials maintain a 100% stone formation rate across all tested water–solid ratios. This indicates that cementitious materials are more sensitive to water–solid ratio changes, exhibiting a more pronounced decline in stone formation rate.

### 3.4. Analysis of Compressive Strength of Hydration Products

Reference samples were prepared using original residue, slag, and NaOH under the same material proportioning conditions, and each sample was labeled as Co-20, Co-40, Co-50, Co-60, Co-70, and Co-80, respectively.

**Figure 10:**
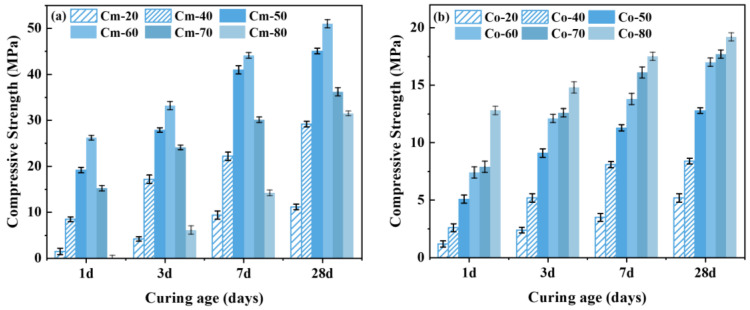
Effect of slag dosing on the compressive strength of grouting materials: (**a**) experimental group; (**b**) control group.

The variation trend of the compressive strength of the grouting material with different slag dosages is shown in Figure 10. The grouting material prepared from alkaline-thermal activated shield sludge and slag has excellent compressive strength. Comparing Figure 10a,b, it can be seen that the compressive strength of the grouting material prepared from unactivated slag is lower, and its 28-day compressive strength is only about 20 MPa at the highest, which is related to the weak hydration reaction activity of the shield sludge; in contrast, the compressive strength of the grouting material prepared from alkali-thermal activated slag is much higher than that of the control group, and its 28-day compressive strength can reach up to 51 MPa at the highest. The results indicate that alkaline-thermal activation is an effective method of slag activation, which can significantly improve its hydration reaction activity.

### 3.5. Hydration Product Analysis

#### 3.5.1. X-Ray Diffractometer Analysis

**Figure 11:**
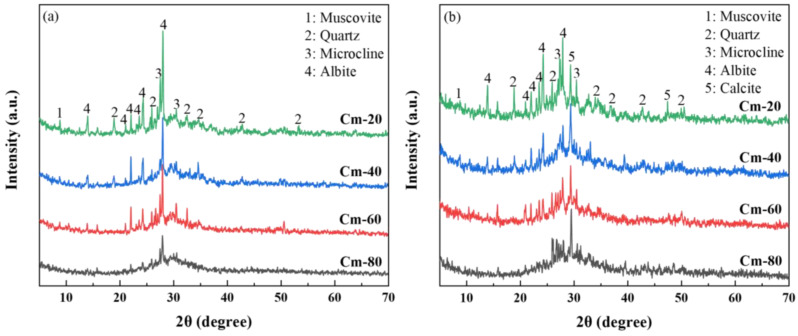
XRD patterns of hydration products in grouting materials cured for (**a**) 1 day and (**b**) 28 days.

Figure 11 presents the XRD patterns of specimens Cm-20, Cm-40, Cm-60, and Cm-80 after 1 and 28 days of hydration. A comparison between the 1-day and 28-day hydration periods reveals a notable reduction in the intensity of diffraction peaks for mica (PDF# 73-1661), quartz (PDF# 79-1906), microcline (PDF# 26-0911), and feldspar (PDF# 80-1094) at 28 days. These peaks correspond to residual mineral phases originally present in the activated slag. Additionally, a diffuse hump in the 25–35° (2θ) range was consistently observed, which is indicative of the formation of C-A-S-H gel [48]. This gel is recognized as the primary hydration product, playing a pivotal role in the development of the material’s compressive strength. After 1 day of hydration, these mineral phases exhibited distinct diffraction peaks across all specimens. As the shield sludge content in the samples increased, the relative proportion of activated slag decreased, resulting in a gradual attenuation of these diffraction peaks. This inverse relationship suggests that the dilution effect of shield sludge addition diminishes the visibility of these phases.

This reduction indicates partial hydration of these phases over time. However, due to the high crystallinity and stable structural nature of these phases, significant diffraction peaks were still detectable at 28 days. Furthermore, the appearance of calcite peaks in the 28-day specimens suggests carbonation, likely due to the interaction between the hydration products and atmospheric CO_2_ during the curing process. The observed variations in compressive strength among the specimens are closely linked to differences in C-A-S-H gel formation, influenced by the degree of shield sludge incorporation [49]. The results underscore that shield sludge addition significantly affects the compressive strength of the material, highlighting the critical importance of C-A-S-H gel as a determinant of the mechanical properties in the system [50].

**Figure 12:**
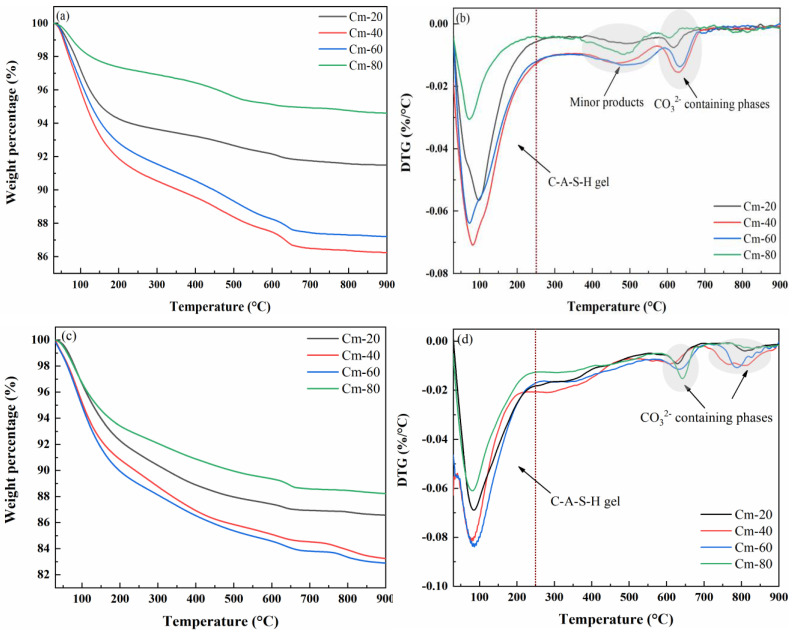
TG/DTG curves of pastes with different Cm contents cured for 1 (**a**,**b**) and 28 days (**c**,**d**).

The TG-DTG curves of pastes with Cm-20, Cm-40, Cm-60, and Cm-80 contents cured for 1 and 28 days are shown in Figure 12a–d. From the curves, it can be seen that the weight loss of the specimens is mainly concentrated in the range of 30–250 °C, which mainly corresponds to the dehydration of C-A-S-H gels. The weight loss of C-A-S-H gel in the hydrated specimens with different shield sludge dosages was obviously different, indicating that the shield sludge dosage has an important influence on the amount of this product generated [51]. A weak weight loss peak was observed in the range of 350–550 °C when the sample was hydrated for 1 day, which disappeared after the hydration time was extended to 28 days. According to the relevant literature, the weight loss peak may be related to hydrotalcite-like compounds [52]. Calcium silicates, such as tricalcium silicate (C_3_S) and dicalcium silicate (C_2_S), are the main anhydrous phases in cement. During hydration, they react with water to form calcium silicate hydrate (C-S-H), which is the principal product responsible for strength development. The weight loss observed in the thermogravimetric curve between 300–700 °C primarily corresponds to the decomposition of C-S-H [9,53].

#### 3.5.2. Hydration Reaction Kinetics of Hydration Products

**Figure 13:**
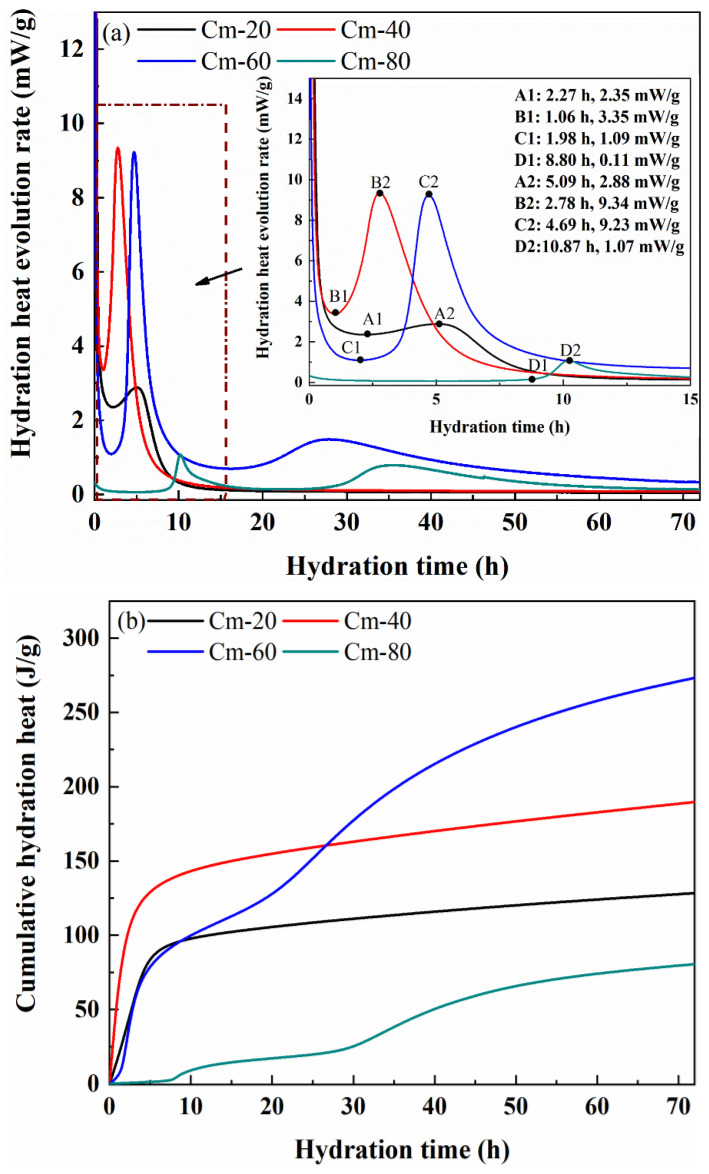
(**a**) Hydration exothermic rate curves and (**b**) cumulative exothermic curves of specimens with different slag dosages.

Figure 13 shows the cumulative exothermic curves of specimens Cm-20, Cm-40, Cm-60, and Cm-80. It can be seen that the cumulative exotherm of sample Cm-80 hydrated for 72 h is the lowest, which is only 80.46 J/g. This result shows that only a small amount of hydration products is generated in the early stage of the hydration reaction, which is the main reason for the weak compressive strength of Cm-80 in the early stage of hydration. Although the exothermic rate of hydration of sample Cm-20 is very fast in the first 5 h, the cumulative exothermic amount at 72 h is only 128.63 J/g due to the low content of shield sludge. The cumulative exothermic amount of sample Cm-40 is higher than that of Cm-60 in the first 24 h. However, at 72 h, Cm-40’s cumulative exothermic amount (189.14 J/g) is significantly lower than that of Cm-60 (273.97 J/g). This indicates that although Cm-40 may have a faster initial reaction due to the release of OH^−^ and active species (Si^4+^, Al^3+^) from the shield sludge, Cm-60 sustains its hydration over a longer period due to the higher slag content [54,55]. The slag provides a continuous supply of reactive aluminosilicate phases, supporting prolonged pozzolanic and hydration reactions and enabling the generation of more hydration products at later stages, thereby contributing to the superior compressive strength of Cm-60.

#### 3.5.3. Microstructural Analysis of Hydration Products

**Figure 14:**
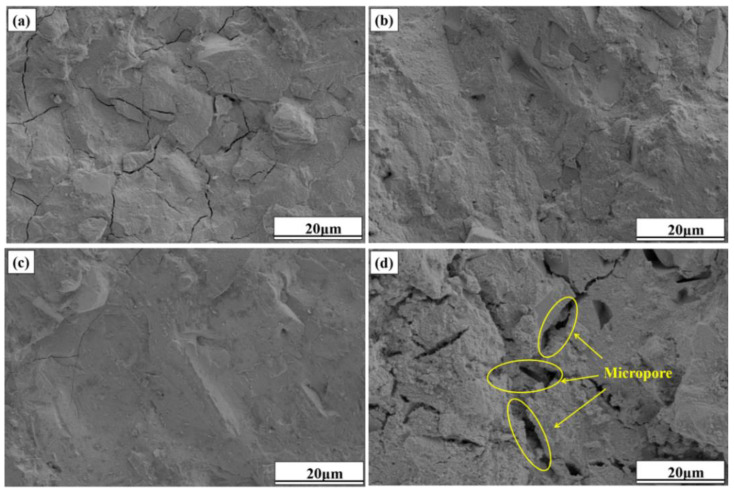
SEM images of specimens (**a**) Cm-20, (**b**) Cm-40, (**c**) Cm-60, and (**d**) Cm-80 after 28 days of conservation.

The SEM images of specimens Cm-20, Cm-40, Cm-60, and Cm-80 after 28 days of hydration are shown in Figure 14. When the ratio of activated slag to slag was controlled at 80%:20% (Figure 14a), a large number of microcracks could be clearly observed in specimen Cm-20. Microcracks are often found in cementitious materials with a high content of alkaline activators, mainly due to internal stresses caused by rapid hydration. In this study, activated slag acts as the main alkaline source [56]. Excessive activated slag increases the concentration of free alkali ions in the pore solution, which accelerates hydration and causes local volume changes. These changes generate internal stress, leading to microcrack formation in the matrix. At an activated slag-to-slag ratio of 40%:60% (Figure 14c), a substantial quantity of C-A-S-H gel is formed, effectively enveloping and bonding the raw material particles into a cohesive matrix. This results in specimen Cm-60 exhibiting a denser and more homogeneous microstructure compared to other specimens, thereby explaining its superior compressive strength. However, when the ratio of activated residue to shield sludge was controlled at 20%:80% (Figure 14d), the denseness of the microstructure of specimen Cm-80 was significantly reduced, with a large number of voids distributed in it and a rougher overall structure. According to the analysis results of thermogravimetric and hydration heat tests, when the proportion of activated slag in the system is too low, the hydration reaction activity of slag cannot be fully stimulated, and the generation of C-A-S-H gel is less, so the raw material particles cannot be gelled into a dense whole. This is also the reason for the lower compressive strength of specimen Cm-80 [57,58].

## 4. Conclusions

This study successfully developed an environmentally sustainable grouting material from shield sludge through NaOH hydrothermal activation. The principal findings are as follows:

1. Activation mechanism: NaOH hydrothermal treatment significantly enhanced the reactivity of shield sludge by decomposing quartz and partially disrupting the crystalline structures of sodium feldspar, muscovite, and microcline. The optimal activation conditions were determined to be 20 wt% NaOH, 200 °C, and 6 h, yielding amorphous sodium aluminosilicate.

2. Geopolymer synthesis: The combination of activated shield sludge and slag facilitated the formation of geopolymers with enhanced compressive strength. The presence of slag accelerated hydration, resulting in compressive strengths of 26.35 MPa, 43.40 MPa, and 51.08 MPa at 1, 7, and 28 days, respectively, when the slag content reached 60 wt%.

3. Commercial feasibility: Despite the promising mechanical properties of the geopolymer as a grouting material, commercialization faces challenges such as the high cost of slag activation and the need for specialized equipment. These barriers could be mitigated by employing more cost-effective activators, such as Na_2_CO_3_ or Ca(OH)_2_, and reducing slag particle size to lower activation temperatures and reaction times. Future research should prioritize the evaluation of long-term durability and environmental performance under varied operational conditions.

## Figures and Tables

**Table 1:** Chemical composition of soil samples (weight%).

Chemical Constitution	SiO_2_	Al_2_O_3_	CaO	Fe_2_O_3_	MgO	Na_2_O	K_2_O	SO_3_	TiO_2_
Shield sludge	33.16	16.31	38.86	0.36	8.37	0.71	/	1.25	0.67
Composite Portland cement	25.46	11.98	49.60	3.72	3.61	/	0.91	3.46	/

**Table 2:** Bleeding rate and stone rate of grouting materials under different water–solid ratios, %.

Injecting Paste Material	Performance Index	Water–Solid Ratio			
		0.4	0.5	0.6	0.7
Muck foundation grouting material	Bleeding rate (%)	-	-	-	-
	Stone rate (%)	100	100	100	100
Cement-based grouting material	Bleeding rate (%)	-	-	4.32	8.75
	Stone rate (%)	-	-	95.68	91.25

## Data Availability

The original contributions presented in this study are included in the article. Further inquiries can be directed to the corresponding authors.

## References

[1] Min F., Dai J., Zhang N., Ma J., Zhang L., Li B. (2024). Investigation into strength development mechanism of lime-modified dehydrated clay of slurry shield tunneling operation. Constr. Build. Mater..

[2] Zhu T., Huang F., Li S., Ouyang T., Ying J., Zhao H. (2024). Optimization of pre-grouting construction and evaluation of grouting effect in a deeply buried silt-filled shield tunnel. Tunn. Undergr. Space Technol..

[3] Zhao H., Wang X., Zhang X., Zhou Y., Wang T., Xue Y. (2024). Preparation of high-strength ceramsite via co-sintering of shield tunnel muck and steel slag: Correlation investigation on phase composition and particle strength. Constr. Build. Mater..

[4] Jiao N., Wan X., Ding J., Zhang X., Xue C. (2024). Mechanical properties and microstructure of lime-treated shield tunnel muck improved with carbide slag and soda residue. Constr. Build. Mater..

[5] Lei J., Yang Y., Chen X. (2024). Mechanics and permeability properties of ecological concrete mixed with recycled engineering muck particles. J. Build. Eng..

[6] Liu K., Pei H., Liang X., Zhao W., Dai G. (2024). Characteristics and modification mechanism of recycled waste silty clay slurry as the shield slag conditioner. Constr. Build. Mater..

[7] Nakao K., Shiina M., Inazumi S. (2024). Assessment of plasticity of muddy soil for earth pressure balance shield tunneling. Tunn. Undergr. Space Technol..

[8] Lee H., Kim H.K., Hwang B., Yoon Y., Choi H. (2024). Coupled DEM-FDM numerical model for EPB shield tunnelling simulation with foam conditioning. Tunn. Undergr. Space Technol..

[9] Elbaz K., Zhou A., Shen S.-L. (2023). Deep reinforcement learning approach to optimize the driving performance of shield tunnelling machines. Tunn. Undergr. Space Technol..

[10] Wu Z., Ye C., Cao F. (2024). Performance and Microstructure of Grouting Materials Made from Shield Muck. Materials.

[11] Ni Z., Wang S., Zheng X., Qi C. (2024). Application of geopolymer in synchronous grouting for reusing of the shield muck in silty clay layer. Constr. Build. Mater..

[12] Chen X., Yu J., Yu F., Pan J., Li S. (2024). The Role of a New Stabilizer in Enhancing the Mechanical Performance of Construction Residue Soils. Materials.

[13] Fang Y., Yao Y., Wang J., Li B., Dou L., Wei L., Zhuo B., Zhang W., Hu X. (2024). Effective dewatering and resourceful utilization of high-viscosity waste slurry through magnetic flocculation. Constr. Build. Mater..

[14] Liu Z., Wu S., Zhou A., Sun X., Xu H., Dong S. (2023). New insight into the additives in preparation and reduction of shield slurry. Sci. Rep..

[15] Yang F., Cao T., Zhang T., Hu J., Wang X., Ding Z., Wu Z. (2023). An Implementation Framework for On-Site Shield Spoil Utilization—A Case Study of a Metro Project. Sustainability.

[16] Gong C.-j., Xie C.-r., Lin Z.-q., Xie D.-w., Zhou Z. (2023). Ground deformation prediction induced by shield tunnelling considering existing multi-story buildings. J. Cent. South Univ..

[17] Zhang K., Zhang Z., Chen H., Hu Z. (2024). Experimental investigation on rheological properties and diffusion-related performance of clay shock slurry for excavation gap filling during shield tunnelling. Tunn. Undergr. Space Technol..

[18] Zhao X., Yang T., Yu Z., Zong Z., Li J. (2024). Study on the Unconfined Compressive Strength Property and Mechanism of Soda Residue Soil. Geotech. Geol. Eng..

[19] Cheng Y., Liu X. (2024). Research on the Pressure Distribution Law of Synchronous Grouting in Shield Tunnels and the Force on Segments. Buildings.

[20] Fan Y., Gao Y., Tao W., Huang S. (2024). Study on the Reuse of Shield Mud from Clay Stratum in Synchronous Grouting Slurry. Buildings.

[21] Zhou Z., Geng J., Jin C., Liu G., Xia Z. (2024). Influence of Residue Soil on the Properties of Fly Ash–Slag-Based Geopolymer Materials for 3D Printing. Materials.

[22] Oggeri C., Fenoglio T.M., Vinai R. (2014). Tunnel spoil classification and applicability of lime addition in weak formations for muck reuse. Tunn. Undergr. Space Technol..

[23] Yu H., Joshi P., Lau C., Ng K. (2024). Novel application of sustainable coal-derived char in cement soil stabilization. Constr. Build. Mater..

[24] Tuncdemir H., Bilgin N., Copur H., Balci C. (2008). Control of rock cutting efficiency by muck size. Int. J. Rock Mech. Min. Sci..

[25] Zheng Y., Xi X., Liu H., Du C., Lu H. (2024). A review: Enhanced performance of recycled cement and CO2 emission reduction effects through thermal activation and nanosilica incorporation. Constr. Build. Mater..

[26] Zhang C., Liu J., Zhang S., Kong X. (2024). Mechanical properties of polymer modified mortars using polymer latexes with varied glass transition temperature and surface charges. Cem. Concr. Compos..

[27] Basquiroto de Souza F., Kai D., Pang S.D. (2024). Nacre-inspired geopolymer cement composite with high flexural strength. Cem. Concr. Compos..

[28] Chen C., Liu H., Zhang Y., Gu G., Hu J. (2024). Micro-assessment of heavy metal immobilization within alkali-activated copper tailings-slag geopolymer. Cem. Concr. Compos..

[29] Zhou H., Li H., Wang Z., Yan D., Wang W., Zhang G., Cheng Z., Sun S., Wang M. (2024). Experimental investigation on the anti-detonation performance of composite structure containing foam geopolymer backfill material. Defence Technol..

[30] Zhang R., Li F., Zhou S., Hou Y. (2023). Carbon nanofiber dispersion in alkali solution and its reinforcement of alkali-activated volcanic ash-based geopolymers. J. Clean. Prod..

[31] Maaze M.R., Shrivastava S. (2023). Development of framework in the selection and reuse of concrete waste and brick waste powder as pozzolanic material in cement concrete application using analytical hierarchy process technique. Constr. Build. Mater..

[32] Zhao L., Wang C., Guo C., Ma X., Li Z., Du X., Jiang W. (2023). Properties of Low-Exothermic polymer grouting materials and its application on highway. Constr. Build. Mater..

[33] Yang C., Shen S.-L., Hou D.-W., Liao S.-M., Yuan D.-J. (2018). Material properties of the seal gasket for shield tunnels: A review. Constr. Build. Mater..

[34] (2021). Test Method of Cement Mortar Strength (ISO Method).

[35] (2023). Common Portland Cement.

[36] Nanda R.P., Priya N., Kumar A. (2025). Enhancement of strength of soft soil utilizing construction and demolition wastes reinforced with Recron-3s fibres. J. Clean. Prod..

[37] Xu J., Zhang M., Lu J., Wang K., Yang F., Chen S., Xu F. (2023). The Influence Mechanism of Molar Ratio on the Performance of Phosphogypsum-Modified Geopolymer Material. Coatings.

[38] Perumal P., Niu H., Kiventerä J., Kinnunen P., Illikainen M. (2020). Upcycling of mechanically treated silicate mine tailings as alkali activated binders. Miner. Eng..

[39] Jeyaprakash R.K.R., Surehali S., Simon A., Han T., Kumar A., Neithalath N. (2024). Early-age reactivity and strength development in high volume mine tailings-based alkali activated binders and their application potential. Miner. Eng..

[40] Cacciuttolo C., Atencio E. (2022). An Alternative Technology to Obtain Dewatered Mine Tailings: Safe and Control Environmental Management of Filtered and Thickened Copper Mine Tailings in Chile. Minerals.

[41] Jiao N., Ding J., Wan X., Gao M., Xue C. (2024). Mechanical properties and micro-mechanism of improved shield tunnel muck with phosphogypsum and lime. Constr. Build. Mater..

[42] Taqa A.A., Al-Ansari M., Taha R., Senouci A., Al-Marwani H.A., Al-Zubi G.M., Mohsen M.O. (2021). Characterization of TBM Muck for Construction Applications. Appl. Sci..

[43] Luo X., Huang L., Yan L., Li Y., Wei L., Chen Z., Qu Y. (2024). Preparation of geopolymers from thermally activated lithium slag: Activity enhancement and microstructure. J. Build. Eng..

[44] Liu Q., Cui M., Li X., Wang J., Wang Z., Li L., Lyu X. (2022). Alkali-hydrothermal activation of mine tailings to prepare one-part geopolymer: Activation mechanism, workability, strength, and hydration reaction. Ceram. Int..

[45] Maruyama I., Meawad A., Kondo T., Sawada S., Halodova P., Fedorikova A., Ohkubo T., Murakami K., Igari T., Rodriguez E.T. (2023). Radiation-induced alteration of sandstone concrete aggregate. J. Nucl. Mater..

[46] Duhme R., Rasanavaneethan R., Pakianathan L., Herud A. (2016). Theoretical basis of slurry shield excavation management systems. Tunn. Undergr. Space Technol..

[47] Naqi A., Otsubo M., Kuwano R., Nagatani H., Kawano K., Liu W. (2025). Influence of length and configurations of stirring rods on the mixing of cohesionless granular particles in earth pressure balance shields. Powder Technol..

[48] Hu Y., Yin S., Li K., Han B., Zhang B. (2023). Formation mechanism and thermal decomposition properties of hydration products of superfine tailings cemented paste backfill. Arab. J. Chem..

[49] Li C., Zhang N., Zhang J., Song S., Zhang Y. (2022). C-A-S-H Gel and Pore Structure Characteristics of Alkali-Activated Red Mud–Iron Tailings Cementitious Mortar. Materials.

[50] Ahmad M.R., Medepalli S., Wang T., Dai J.-G., Zheng Y., Ishida T. (2024). Effect of alkali-hydroxide on hydration kinetics and microstructure of high-volume fly ash blended cement pastes. Cem. Concr. Res..

[51] Qing L., Shaokang S., Zhen J., Junxiang W., Xianjun L. (2021). Effect of CaO on hydration properties of one-part alkali-activated material prepared from tailings through alkaline hydrothermal activation. Constr. Build. Mater..

[52] Jallow A., Ou C.-Y., Lim A. (2019). Three-dimensional numerical study of long-term settlement induced in shield tunneling. Tunn. Undergr. Space Technol..

[53] Vaughn J.S., Marple M.A.T., Mason H.E. (2024). Mechanochemical formation of highly stable amorphous calcium carbonate in calcium silicates: Potential long-term storage of CO2 in cement. Cem. Concr. Res..

[54] Shu X., Jiang Y., Zhao Y., Xu Z., Shen M., Zhong X. (2023). Superimposed hydration exothermic model of cement slurry considering different reaction rates of various active substances. Constr. Build. Mater..

[55] Baltakys K., Dambrauskas T., Rubinaite D., Siauciunas R., Grineviciene A. (2021). Formation and hydration of eco-friendly cement using industrial wastes as raw materials. Sci. Rep..

[56] Liu Q., Li X., Cui M., Wang J., Lyu X. (2021). Preparation of eco-friendly one-part geopolymers from gold mine tailings by alkaline hydrothermal activation. J. Clean. Prod..

[57] Ouyang S., Huang Y., Zhou N., Li J., Gao H., Guo Y. (2022). Experiment on hydration exothermic characteristics and hydration mechanism of sand-based cemented paste backfill materials. Constr. Build. Mater..

[58] Ballim Y., Graham P.C. (2009). The effects of supplementary cementing materials in modifying the heat of hydration of concrete. Mater. Struct..

